# Preoperative metaphyseal cancellous bone density is associated with intraoperative conversion to stemmed total shoulder arthroplasty

**DOI:** 10.1016/j.xrrt.2023.01.009

**Published:** 2023-02-07

**Authors:** James M. Gregory, Jacob Siahaan, Manuel Urvoy

**Affiliations:** a Department of Orthopedic Surgery, University of Texas Health Science Center at Houston, McGovern Medical School, Houston, TX, USA; b IMASCAP, Plouzane, France

**Keywords:** Metaphyseal bone density, Stemless, Total shoulder arthroplasty, Humerus, Preoperative planning

## Abstract

**Background:**

Methods to determine whether a stemless humeral component is appropriate for anatomic total shoulder arthroplasty are varied and often subjective. Objective preoperative data regarding metaphyseal bone quality may help guide surgical decision-making. This study sought to evaluate preoperative proximal humeral bone quality and determine whether it is predictive of intraoperative conversion to a stemmed humeral component.

**Methods:**

Consecutive patients who underwent primary anatomic total shoulder arthroplasty from a single-surgeon practice were enrolled. All patients received a preoperative computed tomography (CT) scan for surgical templating purposes. The exclusion criteria were lack of a preoperative CT scan, preoperative plan for a stemmed component, and intraoperative conversion to a stem for a reason other than bone quality (ie, fracture). Preoperative CT scans were analyzed with an automated templating software. Cortical index and thickness were calculated, and bone density of the proximal diaphysis, cancellous metaphysis, and cortical metaphysis was obtained by averaging Hounsfield units (HU) across anatomically defined regions using a previously validated technique. The decision to convert to a stemmed humeral component was made intraoperatively based on a lack of stability of the trial stemless component. Bone quality measurements were compared between stemless and stemmed groups. An exact logistic regression was used incorporating gender and age.

**Results:**

A total of 79 patients who underwent primary anatomic total shoulder arthroplasty were included in this study. Of these patients, 6 underwent intraoperative conversion to a stemmed humeral component (7.6%). There was no significant difference between cohorts in terms of cortical index and bone density within the proximal diaphysis and cortical metaphysis. On univariate analysis, cortical thickness, metaphyseal cancellous bone density, and gender were significantly different between groups. Patients receiving a stem had significantly lower metaphyseal cancellous bone density than those receiving stemless components (5.5 ± 11.2 HU vs. 47.6 ± 29.4 HU, *P*<.001). All patients converted to stems were female individuals (*P* = .01) and had metaphyseal cancellous bone density less than 20 HU (*P*<.001).

**Conclusions:**

Metaphyseal cancellous bone density can be calculated on preoperative CT scans and is associated with intraoperative conversion to a stemmed humeral component in anatomic shoulder arthroplasty. A threshold of 20 HU can be used to predict which patients are more likely to require stemmed components.

Advances in implant design have led to shorter humeral stem lengths in anatomic total shoulder arthroplasty. Initial humeral stems were monoblock, with no modularity, and very little ability to reproduce the normal center of rotation of the humeral head. Subsequent generations of humeral implants have increased modularity, offset, and provided variable options in stem size and stem length. These changes have allowed better adaptation to humeral anatomy and restoration of more normal shoulder kinematics.^3^ Most recently, stemless humeral components have become more popular in anatomic total shoulder arthroplasty. Proposed benefits included a better ability to reproduce the center of rotation of the humeral head, improved stress loading of proximal humeral bone stock, and ease of revisability.^3^

Long-term stability is achieved in press-fit humeral components once osseointegration occurs between living bone and the implant itself. This process is thought to occur in approximately 3 months.^1^ As a result, initial fixation is imperative to minimize micromotion and allow this osseointegration to occur. Traditional diaphyseally engaging stems gain fixation through diaphyseal reaming to judge stem size, followed by fixation in the meta-diaphyseal region of the proximal humerus. Depending on stem geometry and technique, the stem may abut cortical bone in an attempt to maximize initial stability. On the other hand, stemless humeral components rely on fixation through direct contact with the metaphyseal cancellous bone. No diaphyseal fixation occurs, and no cortical contact is sought. Because cancellous bone density is less than cortical bone density, stemless humeral components have been recommended for patients who “good quality” metaphyseal bone only.

To this point, the determination of what constitutes adequate metaphyseal bone stock for stemless anatomic shoulder arthroplasty is subjective and qualitative. Quantitative methods to evaluate bone density exist, and are used in other parts of the body, but not commonly in shoulder arthroplasty.^5^^,^^6^^,^^8^, ^9^, ^10^, ^11^ The prevalence of osteoporosis across demographic cohorts has led some surgeons avoid stemless components in patients based on age or gender. Other surgeons rely on subjective evaluation of preoperative imaging to determine bone quality. Finally, some surgeons rely on intraoperative assessment of bone quality using manual palpation of the cut surface of the humerus (the so-called “thumb test”), or stability of a trial stemless component.^4^^,^^8^ Although the outcomes of stemless anatomic total shoulder arthroplasty using these qualitative techniques is excellent,^4^^,^^7^^,^^8^ we believe that quantitative measurement of metaphyseal bone density would be valuable. We hypothesize that it may improve accuracy of preoperative planning, and may be beneficial for surgical decision-making.

In this study, we sought to quantitatively evaluate proximal humeral bone quality in patients undergoing anatomic total shoulder arthroplasty, and determine whether it is predictive of intraoperative conversion to a stemmed humeral component.

## Methods

### Study design

Local institutional review board approval was obtained prior to initiation of this retrospective cohort study. Consecutive patients who underwent primary anatomic total shoulder arthroplasty from a single-surgeon referral practice were enrolled. All patients received a preoperative computed tomography (CT) scan for surgical templating purposes. The exclusion criteria were lack of a preoperative CT scan, preoperative plan for a stemmed component, and intraoperative conversion to a stem for a reason other than bone quality (ie, fracture). All patients were planned and templated to receive a stemless anatomic humeral component (Simpliciti; Tornier, Bloomington, MN, USA). After the humeral cut was performed at the anatomic neck, a stemless anatomic humeral trial component was placed, followed by a head protector. The glenoid was prepared, and a final glenoid component was placed. When attention was turned back to the humerus, the head protector was removed. If the trial component was stable, then a final stemless component was inserted. If the trial component was grossly mobile, then the decision was made to convert to a stemmed humeral component.

### Radiographic analysis

Preoperative CT scans were analyzed with an automated templating software. All measurements were made using Glenosys planning software (Imascap, Brest, France). The metaphysis was defined as the region between the anatomic neck and the surgical neck. Cortical index and thickness were calculated, and bone densities of the proximal diaphysis, cancellous metaphysis, and cortical metaphysis were obtained by averaging Hounsfield units (HU) across anatomically defined regions using previously validated techniques.^2^

### Statistical analysis

Preoperative bone quality measurements were compared between stemless and stemmed groups. Fisher exact tests were used for categorical variables and Mann-Whitney U tests were used for continuous variables. Calculations were performed on STATA 17.

## Results

**Figure 1:**
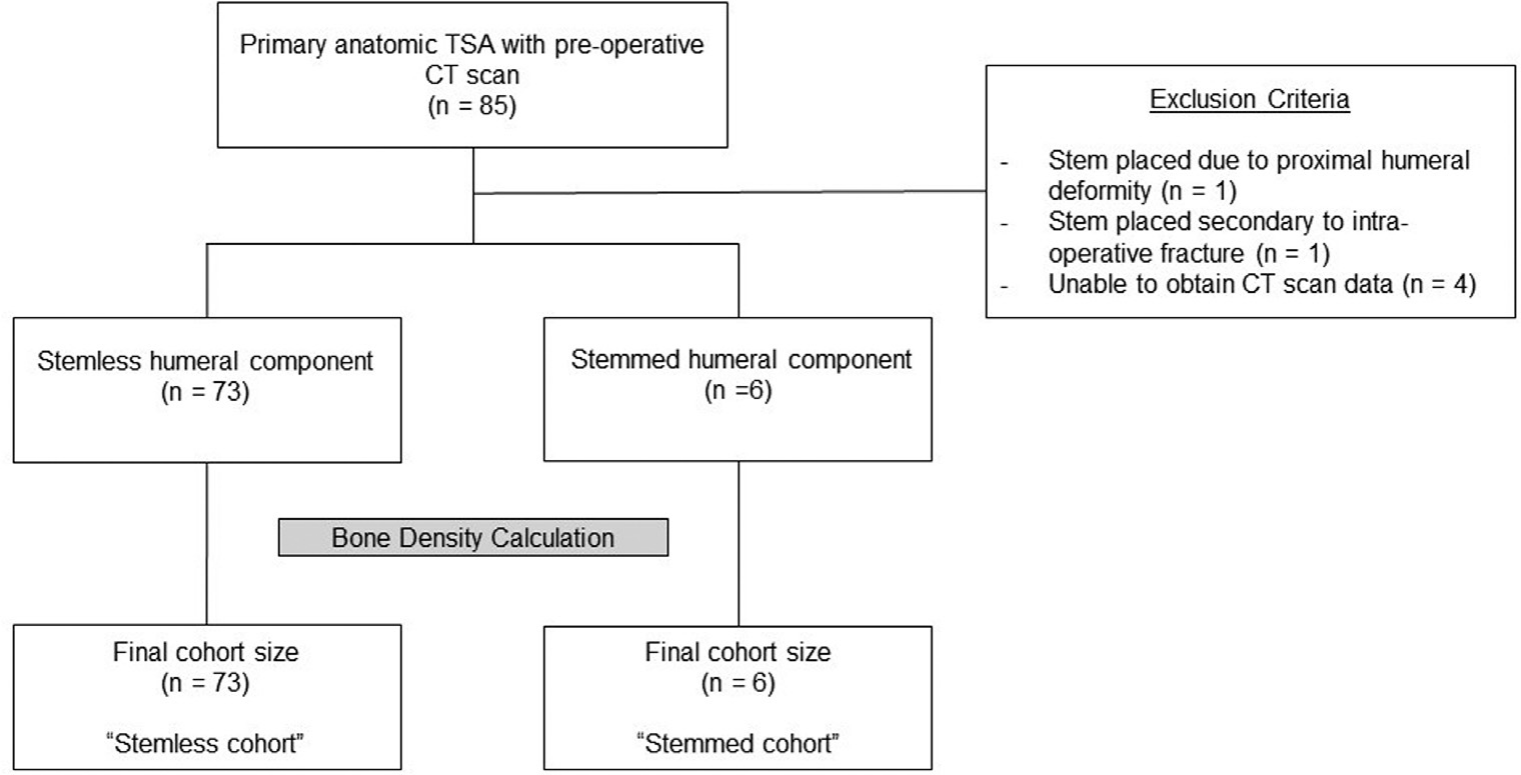
Flowchart of patient enrollment, exclusions, and data analysis. *TSA*, total shoulder arthroplasty; *CT*, computed tomography.

A total of 85 consecutive patients who underwent primary anatomic total shoulder arthroplasty were screened for inclusion in the study. Six patients were excluded prior to data analysis. Four of these patients had CT scans that were unable to be analyzed. One patient had placed placement of a humeral stem secondary to proximal humerus bone deformity. Another patient with osteogenesis imperfecta received a humeral stem secondary to an intraoperative proximal humerus fracture. Patient enrollment and analysis is shown in Figure 1. A total of 79 patients met inclusion criteria and were included in the study. Of these patients, 6 underwent intraoperative conversion to a stemmed humeral component (7.6%).

Based on univariate analysis, which is shown in Table I, Tingart cortical thickness was significantly lower in the stemmed cohort (stem: 3.2 ± 0.3 mm; stemless: 3.8 ± 0.7 mm, *P* = .012). All patients in the stemmed cohort were female and had metaphyseal cancellous bone density less than 20 HU. Patients in the stemmed cohort had a significantly lower metaphyseal cancellous bone density (stem: 5.5 ± 11.2 HU; stemless: 47.6 ± 29.4 HU, *P* < .001). Since all patient who required stems had densities of less than 20 HU, this value was subsequently set to separate high and low metaphyseal cancellous bone density. More patients in the stemless cohort had low metaphyseal cancellous bone density (stem: 100%, n = 6/6; stemless: 21.5%, n = 15/73, *P* < .001). Gender breakdown was significantly different between cohorts (female gender – stem: 100%, n = 6/6; stemless: 43.8%, n = 32/73, *P* = .01). Age and other measurements of proximal humerus bone density were not significantly different between cohorts.

**Table I:** Univariate analysis of variables between stemmed and stemless groups.

Variable	Stem (n = 6)	Stemless (n = 73)	*P* value
Gender			.01∗
Male	0	41 (56.2%)	
Female	6 (100%)	32 (43.8%)	
Age (yr)	67.6 ± 9.0	73.2 ± 4.8	.07
Tingart cortical thickness (mm)	3.2 ± 0.3	3.8 ± 0.7	.012∗
Metaphyseal cancellous bone density (HU)	5.5 ± 11.2	47.6 ± 29.4	<.001∗
High (above 20)	0	58 (79.5%)	<.001∗
Low (20 and below)	6 (100%)	15 (21.5%)	<.001∗
Gianotti cortical index	0.6 ± 0.1	0.6 ± 0.1	.79
Proximal diaphysis bone density (HU)	1054.6 ± 111	926.3 ± 180	.12
Metaphysis cortical bone density (HU)	559.5 ± 80.5	502.0 ± 91.7	.1

*HU*, Hounsfield units; *mm*, millimeters.

∗ *P* < .05.

## Discussion

The results of this study show that metaphyseal cancellous bone density can be quantified and calculated on preoperative CT scans and is associated with intraoperative conversion to a humeral stem. A value less than 20 HU has a 17.8 times likelihood of needing a stemmed component. This 3-dimensional (3D) CT measurement of metaphyseal cancellous bone density has previously been correlated with both validated 2-dimensional measures of proximal humerus bone density, in addition to intraoperative assessment of bone density using a diaphyseal sounder.^2^ In that study, other 3D measurements of proximal humeral bone density were also calculated, but were not found to be as correlated. Similarly, in our study, although other measurements of proximal humeral bone density were performed, none were found to be as strongly correlated with stem usage. This is logical, as stemless components rely on metaphyseal cancellous fixation for initial stability. If metaphyseal cancellous bone density is low, then trial component stability may be more likely compromised. Other measurements of proximal humeral bone density, such as metaphyseal cortical thickness or diaphyseal bone density, may be correlated with poor bone quality overall, but do not directly contribute to the stability of the component itself.

This study involves cases performed by a single surgeon. Conversion to a stem was performed based on stability of the trial humeral component, and therefore these results are based on this method of stability assessment. We believe that assessing stability of the trial component is the most representative way of determining final humeral component stability. Other would necessarily lead to different results. Currently, existing ways of estimating bone quality are largely qualitative and driven by experience. Subjective bone quality measurements such as the “thumb test,” have been found to be divergent among surgeons.^12^ In addition to being unreliable across individuals, thus preventing standardized assessment of bone quality, experience-based measurements make decision-making more complicated for surgeons who may not yet have a large pool of shoulder arthroplasty experience on which to draw. Making decision about bone quality based off of demographic information is similarly inexact. Although rates of osteoporosis increase with age, age was not found to be correlated with use of a stemmed component in our cohort. Even though all patients who received a stemmed component were female, upon logistic regression analysis, gender was no longer significant. Avoiding stemless components in all women would eliminate nearly half of patients in whom a surgeon may desire to use a stemless component.

It is important to note that only a small portion of patients in this study needed intraoperative conversion to a stemmed component secondary to poor bone quality (7.6%). As mentioned, 2 patients received stems for indications other than bone quality and were excluded. In our practice, stemless humeral components are a default for patients undergoing anatomic total shoulder arthroplasty, and no selection bias exists in the cohort of patients selected for inclusion in this study. Put simply, in our data, for the vast majority of patients, bone quality does not limit the use of stemless humeral components. What, then, is the value of calculating preoperative metaphyseal bone quality? This study identifies a 3D measurement of metaphyseal bone quality that is strongly correlated with a clinically relevant end point, that is, use of a stem. This data is important for preoperative planning, surgical decision making, implant management, and may help guide research into topics related to proximal humeral bone quality. As preoperative planning becomes more prevalent in shoulder arthroplasty, automatic calculation of proximal humeral bone quality may help indicate to surgeons whether caution needs to be taken if stemless components are desired. Similarly, if very high metaphyseal bone quality measurements are known preoperatively, less stemmed backup implants may be required, which can improve efficiency and cost savings. Metaphyseal cancellous bone quality can also potentially be used as part of long-term studies of stemless components, to evaluate risk factors for long-term humeral outcomes such as subsidence or bony resorption. Finally, cadaveric measurements of proximal humeral bone density can be used during biomechanical implant testing to better understand force thresholds of implant failure.

This study possesses several strengths. Importantly, this study uses a 3D measurement of bone density across the entire metaphyseal region of the proximal humerus, not at isolated points. The segmentation and calculation of this measurement was done automatically, and requires no surgeon-led input. No special software or additional tests were needed (ie, DEXA screening). A standard templating CT was used, which incorporated 1-mm axial slices. As this was a single surgeon series using a single implant system, there was a consistent technique and indication for stem usage, which decreased variability. The surgical decision-making to use a stem was based off stability of the trial humeral component, and was independent of the measured bone density.

However, the study does have its limitations. Rates of intraoperative conversion to a stem were low. This reflects the fact that stemless components are suitable for the vast majority of patients in our study. As such, a meaningful logistic regression analysis could not be performed. Whenever samples sizes are small, concern exists that a study may be underpowered to detect significant differences between groups (type II error). However, the differences between groups in terms of bone density are significant, which means that type II error is not present for those measurements. Additionally, not all surgeons obtain CT scans preoperatively for planning purposes. Depending on implant system and surgeon preference, other studies such as an magnetic resonance imaging may be utilized. This measurement is not validated for calculation on magnetic resonance imaging. Additionally, as mentioned, this study only looks at the correlation between proximal humeral bone density and intra-operative conversion to a stem. Clinical and radiographic outcomes are outside the scope of this manuscript but are very important. Stratifying long-term outcomes based on preoperative metaphyseal bone density will be an important future direction. However, existing studies have shown success rates of stemless total shoulder arthroplasty that are comparable to stemmed components.^3^^,^^4^^,^^7^^,^^8^ As this study involves only 1 type of stemless humeral component, the metaphyseal bone density at which conversion to a stem becomes common may different. Other designs may be more or less stable at time zero. Consequently, it is important to note that the specific threshold value of 20 HU may be different depending on prosthesis design. Along the same line, this threshold is likely not applicable to stemless reverse humeral components. Stemless reverse humeral components are currently undergoing clinical evaluation in the United States. These components also rely on metaphyseal cancellous fixation, so we hypothesize the metaphyseal bone density would be equally as important. However, the forces seen by reverse shoulder arthroplasty are much different than anatomic shoulder arthroplasty and include a “tip-out” force instead of largely compressive forces. We anticipate a higher threshold of bone quality is needed to support these components.

Regardless, this technique for quantifying metaphyseal cancellous bone density has strong clinical relevance in terms of conversion to a humeral stem. Further study is needed to better understand how metaphyseal cancellous bone density affects longevity of stemless humeral implants, as well as bone adaptations such as stress-shielding seen postoperatively.

## Conclusion

Metaphyseal cancellous bone density can be calculated on preoperative CT scans and is associated with intraoperative conversion to a stemmed humeral component in anatomic shoulder arthroplasty. A threshold of 20 HU can be used to predict which patients are more likely to require stemmed components.

## Disclaimers:

Funding: No funding was disclosed by the authors.

Conflicts of interest: James M. Gregory discloses the following conflicts: Consultant Agreement, Research Support – Stryker Consultant Agreement, Research Support – Arthrex Stock/Stock Options – Sparta Biomedical. Manuel Urvoy discloses the following conflicts: Employment – IMASCAP. The other authors, his immediate families, and any research foundation with which he is affiliated have not received any financial payments or other benefits from any commercial entity related to the subject of this article.
